# Surveillance of Microbiological Environment of Operation Theaters

**DOI:** 10.7759/cureus.20525

**Published:** 2021-12-20

**Authors:** Aparna Shukla, Shilpi Srivastava, Aman Srivastava, Tanushree Srivastava

**Affiliations:** 1 Anaesthesiology, King George's Medical University, Lucknow, IND; 2 Microbiology, Integral Institute of Medical Sciences and Research Centre, Lucknow, IND; 3 Anaesthesiology, Medanta Super Speciality Hospital, Lucknow, IND

**Keywords:** environmental monitoring, operation theaters, pathological microorganism, fumigation, disinfection

## Abstract

Introduction

Control of infections in the operation theater (OT) is of utmost importance. Microbiological surveillance is an effective tool for identifying and controlling infections. The purpose of this study was to investigate the prevalence rate of microorganisms in OTs, to identify the type of microorganisms, and to detect contamination of various surfaces and air of OT.

Methods

OTs were properly cleaned with soap and water. All surfaces were disinfected, followed by fumigation with quaternary ammonium compounds. OTs were kept closed overnight. In the morning, they were opened, and samples were collected, taking all aseptic precautions. The settle plate method was used for air sampling, and the swab method was used for surface sampling. Samples were collected from four surfaces of OTs, i.e., floor, wall, table, and light, and samples of the OT air were also collected and immediately transported to the microbiology laboratory of the institution in sterile conditions.

Result

A total of 1640 swab samples were taken from eight OTs, out of which 487 (29.7%) were found positive for bacterial growth. Most of them were non-pathological microorganisms such as aerobic spore-forming Bacilli and Micrococcus. Among various OTs, septic OT showed the highest bacterial growth (82 positive cultures out of 200). In the surface sampling of various OTs, aerobic spore-forming Bacilli (221/487) was the most common isolate, followed by coagulase-negative Staphylococci (74/487), and Micrococcus (67/487). General surgery, septic, and emergency OTs had maximum air bioload (97, 93, and 91 colony-forming unit (CFU)/M^3^, respectively).

Conclusion

In surface sampling of OTs, it was found that septic OT and general surgery OT were most contaminated where the patient load was high. Among all the surfaces, OT walls and tables were most contaminated with pathogenic microorganisms. The average air bioload of all OTs was ranged between 79 and 97 CFU/M^3^.

## Introduction

Medical science has evolved tremendously since the time of Louis Pasteur who gave the germ theory of disease. Since then, the prime responsibility of the physician has become the prevention and control of various microbes at every place, especially in health care facilities. Hospital-acquired infections are a major cause of morbidity and mortality in the patients coming to hospitals for various reasons [1]. The environment of operation theater (OT) plays a major role in the postoperative recovery of the patients. Infection acquired in OTs leads to increased morbidity and mortality, prolonged length of hospital stay, increased expenditure of patient and hospital [2,3]. Using good theater practice, standard cleaning, and proper sterilization, infections in the OTs can be minimized. The primary objective of our study was to detect if there is any bacterial colonization of indoor air and surfaces of OTs even after cleaning and fumigation. Secondary objectives were to estimate the bacterial colony-forming unit (CFU) rate of indoor air and to identify the type of bacterial contamination of OTs.

## Materials and methods

Study design

This was a prospective study. Ethical committee approval was taken [IEC/IIMS&R/2019/08].

Inclusion criteria

Samples were collected from all OTs on Monday under proper sterile conditions.

Exclusion criteria

If OT was opened before 12 hours, the samples from that OT was not collected. Further, if there was any contamination of samples during collection or transportation, the samples were discarded.

Data collection

Samples were collected for 1 year from eight OTs and processed in the microbiology department. The human subject or any related biological tissue was not included in the study. The division of eight OTs was as follows: OT1: gynecology; OT2: ENT; OT3 &4: general surgery, OT5: septic, OT6: emergency, OT7: obstetrics, and OT8: ophthalmology. Samples were collected from four surfaces (floor, wall, table, and light) and air of OTs. All OTs were properly cleaned with soap and water and thereafter mopped and fumigated with quaternary ammonium compounds. OTs were kept closed overnight. In the morning, OTs were opened, and samples were collected wearing sterile gloves, masks, and sterile gowns. Two sampling procedures were used in this study: the settle plate method was used for air sampling and the swab method was used for surface sampling.

All samples were appropriately labelled and immediately transported to the microbiology laboratory in sterile conditions.

Laboratory diagnosis

Culture

Swabs taken from different sites were inoculated on blood agar, MacConkey agar or cystine-lactose-electrolyte-deficient (CLED) agar and incubated at 37°C for 18-24 hours under aerobic conditions. After incubation of blood agar (for air sample) at 37°C for 18-24 hours under aerobic conditions, the colonies were counted and converted into colony-forming units per cubic meter of air (CFU/M^3^) by using the Omeliansky formula.

N=5a x 104 (bt)^−^^1^

N=colony-forming unit per cubic meter of air (CFU/M^3^) a=number of colonies per petri dish b=surface area of petri dish in cm^2^ t=time exposure (minutes)

Microscopy

A smear was made from a bacterial colony and examined under oil immersion after Gram’s staining. Isolates were identified on the basis of colony morphology, motility, catalase test, coagulase test, oxidase test, and different biochemical tests.

Statistical analysis

Data was analyzed with software IBM Statistical Package for Social Sciences (SPSS) version 23.0 (IBM, Chicago, IL). It was hypothesized that there is no contamination of air or surfaces of OTs. Quantitative data was analyzed by student-t-test and qualitative data by the Chi-square test. P-value less than 0.05 was considered statistically significant.

## Results

A total of 1640 swab samples were taken from eight OTs, out of which 487 (29.7%) were found positive for bacterial growth. Higher growth of non-pathogenic organisms in comparison to pathogenic organisms was found in all OTs (311 and 176, respectively). Septic OT (OT5) showed the highest bacterial growth (82 positive samples out of 200) followed by general surgery (OT4) (78 out of 208). OT8 was least contaminated (46 positive samples out of 204). The difference between sterile and positive swabs and the difference between pathogen and non-pathogen were statistically significant (Table 1).

**Table 1:** Surface sampling of OTs according to sterility and culture positivity (non-pathogen vs pathogen). OT: operation theater *significant **significant (P<0.05 was considered significant)

OT	Total swabs	Number of sterile swabs	Number of positive cultures	P-value	Number of non-pathogen	Number and pathogens	P-value
1	204	151	53	0.000013*	30	23	0.000454**
2	204	157	47		27	20	
3	208	133	75		52	23	
4	208	130	78		53	25	
5	200	118	82		46	36	
6	208	151	57		35	22	
7	204	155	49		35	14	
8	204	158	46		33	13	
Total	1640	1153	487		311	176	

A maximum positive culture was found on the OT table followed by walls (132 and 129, respectively). OT walls were highly contaminated with pathogenic organisms (55 pathogens out of 129) followed by the table (45 pathogens out of 132). The differences between the number of sterile swabs and positive cultures were statistically insignificant. Similarly, there was no significant difference between the numbers of pathogenic and non-pathogenic bacteria (Table 2).

**Table 2:** Contamination status of surfaces of OTs. OT: operation theater *Non-significant

Surfaces	Number of sterile swabs	Number of positive cultures	P-value	Number of non-pathogens	Number of pathogens	P-value
Floor (410)	296	116	*0.3991	116	34	*0.659
Wall (410)	280	129		129	55	
Table (410)	277	132		132	45	
Light (410)	300	110		110	42	
Total (1640)	1153	487		487	176	

In the surface sampling of various OTs, aerobic spore-forming Bacilli (221/487) was the most common isolate, followed by coagulase-negative Staphylococci (74/487), and Micrococcus (67/487) (Table 3).

**Table 3:** Number of organisms colonizing on various surfaces of OTs. OT: operation theater; ASB: aerobic spore-forming Bacilli; CoNS: coagulase-negative Staphylococci

Organisms	Total number of positive swabs on the floor	Total number of positive swabs on the wall	Total number of positive swabs on the table	Total number of positive swabs on the light	Total no of positive swabs on all the surfaces
ASB	60	54	57	50	221
Micrococcus	20	14	21	12	67
Diphtheroids	0	1	1	1	3
Staphylococcus aureus	16	23	11	13	63
CoNS	12	19	25	18	74
Pseudomonas	3	2	4	3	12
Escherichia coli	1	5	2	2	10
Klebsiella	1	1	2	1	5
Acinetobacter	2	9	8	6	25
Enterococcus	1	0	1	3	5
Enterobacter	0	0	0	1	1
Serratia	0	1	0	0	1
Total	116	129	132	110	487

The average air bioload of all OTs was ranged between 79 and 97 CFU/M^3^. The highest air bioload was found in OT3 followed by OT5, OT6, OT4, OT8, OT7, OT1, and OT2 (Table 4). However, the difference was not significant statistically.

**Table 4:** Air bioload (CFU/M3) of OTs. OT: operation theater

OT No.	Air bioload [CFU/M^3^]	P-value
1	81	X=2.528 P=0.9250
2	79	
3	97	
4	88	
5	93	
6	91	
7	86	
8	87	

## Discussion

Microbial contamination of the OT environment can be due to a variety of causes, and it leads to postoperative infections that can prolong hospital stays, cause long-term disability, and increase resistance to antibiotics. It also imposes an additional financial burden on the patients.

In our study, a total of 1640 swab samples were taken from eight OTs, out of which 487 (29.7%) were found positive for bacterial growth. The bacterial count was highest in septic OT (82/200) followed by general surgery (78/208). Ophthalmology and ENT OTs were the least contaminated where the bacterial count was 46 and 49, respectively. The most common isolates were aerobic spore-forming Bacilli (221/487) followed by coagulase-negative Staphylococci (74/487), Micrococcus (67/487), and *Staphylococcus aureus* (63/487). Among the various surfaces of OTs, OT tables were highly contaminated, followed by walls. The average air bioload of all OTs ranged between 79 and 97 CFU/M^3^. The highest air bioload was found in OT3 (97 CFU/M^3^) followed by OT5 (93 CFU/M^3^), OT6 (91 CFU/M^3^), OT4 (88 CFU/M^3^), OT8 (87 CFU/M^3^), OT7 (86 CFU/M^3^), OT1 (8 CFU/M^3^), and OT2 (79 CFU/M^3^).

Najotra et al. collected a total of 4378 samples and out of which only 195 (4.4%) samples were contaminated with pathogenic and non-pathogenic bacteria. The predominant isolates were Bacillus (184) followed by coagulase-negative Staphylococci (17). Though their study surfaces were less infected ,air sampling of various OTs showed air bioload in ranges of 27-133 CFU/M^3^ with least contamination of ophthalmic OT and the highest rate of contamination in general surgery OT [4].

In the study by Kausar et al., a total of 134 swabs out of 184 swabs were found to be positive for bacterial growth and total of 41 air samples out of 43 were found to be positive for bacterial contamination. Average air bioload was 4.4 CFU/M^3 ^to 268.7 CFU/M^3^ with the least CFU/M^3^ being found in ophthalmology OT (4.4-10 CFU/M^3^) and highest in gynecology and obstetrics OT (4.4-268.7 CFU/M^3^). The most predominant microorganism was Bacillus followed by coagulase-negative *S. aureus*. These findings co-related with our study [5].

In the study by Kiranmai and Madhavi, a total of 48 bacterial species were isolated from 111 swab samples from all OTs and ICUs. The highest contaminated surface was OT table as found in our study. Their study showed that OTs had bacterial CFU rate of air varying from 6 to 72 CFU/M^3 ^which is much less than in our study. The most common isolates were Bacillus species 36 (75%) followed by Micrococcus [6].

Deepa et al. did a surveillance of microbiological flora of critical care unit and opined that bioload helps in monitoring the capability of the air filters used in the OTs and also helps in assessing quality and making timely changes in measures that need to be adopted to maintain the air quality in these areas. Therefore, she stressed upon that strengthening surveillance and laboratory capacity will surely enhance infection prevention and control [7].

New WHO recommendations on preoperative measures for surgical site infection preventionalso suggested integral measures such as discontinuation of the practice of hair removal inside the OT and preoperative bathing of patients with soap and water [8].

It was a routine practice in our theaters to fumigate the OTs after cleaning only once a week except under special circumstances. No ultraviolet device was available. Frequent touching of OT tables by the staff without wearing gloves and direct contact with the patients might be the possible cause of highly contaminated OT tables. Therefore, hand hygiene is of utmost importance in preventing cross-infections among staff and patients. The tendency of people leaning against the wall might be one of the reasons of contaminating it.

In our study, septic OT was most infected and it might be because cleaning of these OTs needs special attention as highly infected cases are performed in these OTs, leading to high bacterial load. General surgery OT had the highest patient load and, therefore, should be cleaned and sterilized more frequently and more meticulously. To control airborne contamination of OTs, it is essential to limit unnecessary traffic inside OTs. Further proper ventilation inside the OT with the provision of laminar flow and change of indoor air at least 20-25 times per hour or more depending upon surgical load is also recommended. Bacterial air count is costly but a good indicator for predicting postoperative infections, and it is, therefore, recommended.

Mandatory teaching and training of all OT staff should be included in standard operative procedure of OTs. Some studies suggest that the use of alcohol-based gel drastically reduces infection [9]. In addition, all waste from the OT should be collected in closed airtight containers. Time to time surveillance of the environment of OTs should not be ignored at any cost to assess microbial load and to identify predominant pathogens [10,11].

National Infection Control Guidelines, Draft version 2017 suggested few guidelines for prevention and control of infections inside the OTs. Few highlights of the guidelines suggest that at the beginning of each day a clean damp lint should be used to remove all the dust from all flat surfaces of OT. However, after completion of OT, terminal cleaning of all operating room surfaces, hand washing areas, trolleys, and storage areas should be done irrespective of their usages. For wiping of surfaces, freshly prepared solution of 0.5% chlorine should be used. All waste from the OT should be collected in closed airtight containers. Similarly all soiled equipment should be decontaminated by soaking in 0.5% chlorine solution for 10 minutes. Wet clothes easily get contaminated with microorganisms and hence should be dried completely before use [12].

In this study, we used the settle plate’s method for air sampling and swabbing technique for surface sampling, which are considered as coarse methods and was a significant limitation of our study.

## Conclusions

This study suggested that indoor air and surfaces of OTs were infected mostly by non-pathological microorganisms. Septic OT and general surgery OT had high bacterial growth. Aerobic spore-forming Bacilli were the most common isolate followed by coagulase-negative Staphylococci. Among all the surfaces, OT tables and walls were most contaminated. These surfaces also had the highest count of pathological microorganisms. The average air bioload of all OTs ranged between 79 and 97 CFU/M^3^. Periodic and regular microbiological surveillance of OTs is required to detect and control infection and it is strongly recommended.
